# Graphene and Carbon Nanotubes Fibrous Composite Decorated with PdMg Alloy Nanoparticles with Enhanced Absorption–Desorption Kinetics for Hydrogen Storage Application

**DOI:** 10.3390/nano11112957

**Published:** 2021-11-04

**Authors:** Bassim Arkook, Ahmed Alshahrie, Numan Salah, Mohammad Aslam, Saeed Aissan, Ashwaq Al-Ojeery, Ahmed Al-Ghamdi, Akihisa Inoue, El-Sayed Shalaan

**Affiliations:** 1 Department of Physics, Faculty of Science, King Abdulaziz University, Jeddah 21589, Saudi Arabia; aalshahri@kau.edu.sa (A.A.); saealzahrani@kau.edu.sa (S.A.); aaalaegiry@uj.edu.sa (A.A.-O.); agamdi@kau.edu.sa (A.A.-G.); ainouebmg@yahoo.co.jp (A.I.); 2 Department of Physics and Astronomy, University of California, Riverside, CA 92521, USA; 3 Center of Nanotechnology, King Abdulaziz University, Jeddah 21589, Saudi Arabia; nsalah@kau.edu.sa; 4 Center of Excellence in Environmental Studies, King Abdulaziz University, Jeddah 21589, Saudi Arabia; aslam312@gmail.com; 5 Department of Physics, Faculty of Science, Jeddah University, Jeddah 23890, Saudi Arabia; 6 International Institute of Green Materials, Josai International University, Togane 283-8555, Japan

**Keywords:** hydrogen storage, carbon nanotubes, nanomaterials, thin films

## Abstract

We describe a graphene and fibrous multiwall carbon nanotubes (*f*-MWCNT) composite film prepared by plasma-enhanced chemical vapor deposition for use as a suitable and possible candidate of hydrogen storage materials. A high storage capacity of 5.53 wt% has been obtained with improved kinetics. The addition of binary PdMg alloy nanoparticles to the surface of graphene-fibrous nanotubes composite films raised the storage capacity by 53% compared to the film without PdMg decorated nanoparticles. Additionally, the graphene/*f*-MWCNT composite film decorated with PdMg nanoparticles exhibited an enhanced hydrogen absorption–desorption kinetics. The fibrous structure of the MWCNTs, alongside graphene sheets within the film, creates an enormous active region site for hydrogen reaction. The addition of PdMg nanoparticles enhanced the reaction kinetics due to the catalytic nature of Pd, and increased the hydrogen content due to the high absorption capacity of Mg nanoparticles. The combination of Pd and Mg in a binary alloy nanoparticle enhanced the hydrogen capacity and absorption–desorption kinetics.

## 1. Introduction

Today, hydrogen is considered the next-generation energy carrier for vehicles and fixed engines or power sources [1,2,3,4,5]. Hydrogen exhibits the highest energy density per mass of around 40 kWhkg^−1^. When used as a source of energy, the exhaust product is water only. Before hydrogen can be used in portable applications, it is necessary to find the appropriate technology that is most economical and safe to store hydrogen at the highest possible density. In practice, for a light-duty vehicle, the key for hydrogen storage is how to release the amount of hydrogen that has been stored to allow a driving range of at least 480 km against the vehicle engineering limits of weight, volume, safety, efficiency, and cost—plus, of course, the durability. There are currently some different hydrogen storage technologies that are heavily investigated [6,7,8,9,10,11,12,13]. Unfortunately, all those technologies did not reach the satisfaction conditions for being commercialized. Accordingly, there is a strong demand for innovative technology or new materials that exhibit distinctive and unique properties for hydrogen storage.

In general, hydrogen can be stored in gaseous or liquid form. However, these methods require a significantly large volume and weight, which makes them unsuitable for portable applications. Therefore, storing hydrogen in solid form is a necessary solution, for example, in the transportation sector.

Solid-state hydrogen storage materials can be classified into: i—hydrides (light metal hydrides and complex metal hydrides) [14,15,16,17]; ii—carbon-based [18,19,20,21,22,23,24,25]; iii—chemical hydrogen storage [26,27,28,29,30,31,32,33]; and iv—new advanced materials [34,35,36,37,38,39,40,41]. Among these materials, the hydride is preferred for hydrogen storage due to two facts: large amounts of hydrogen can be stored, and hydrogen absorption and desorption kinetics can be improved by varying the metallic alloy elements or compositions.

On this subject, MgH_2_ has been one of the most considered materials among several high-potential hydride systems due to its high volumetric (110 kg/m^3^) and gravimetric (7.6 wt%) hydrogen storage capacities, as well as its natural abundance and good reversibility. However, the slow hydrogenation kinetics, low hydrogen diffusion coefficient, and surface oxide layer formation limit practical applications. Therefore, several solutions have been proposed to enhance and improve the hydrogen storage properties, including alloying Mg with other elements and catalysts or producing it at the nanoscale [42,43,44].

Accordingly, various transition metals, rare earth metals, and other elements produce magnesium-based metal alloy hydrides for hydrogen storage. In addition, some noble metal catalysts, such as Pd, have been reported to be effective in assisting the hydrogenation of MgH_2_. It was found that during the hydrogenation process, the Pd metal catalyst has a significant effect, causing an increase in the diffusion of hydrogen atoms at the Pd–Mg interface. Many investigations are needed to inspect the properties of the lightweight metal hydrides and tailor their absorption/desorption kinetics.

**Figure 1:**
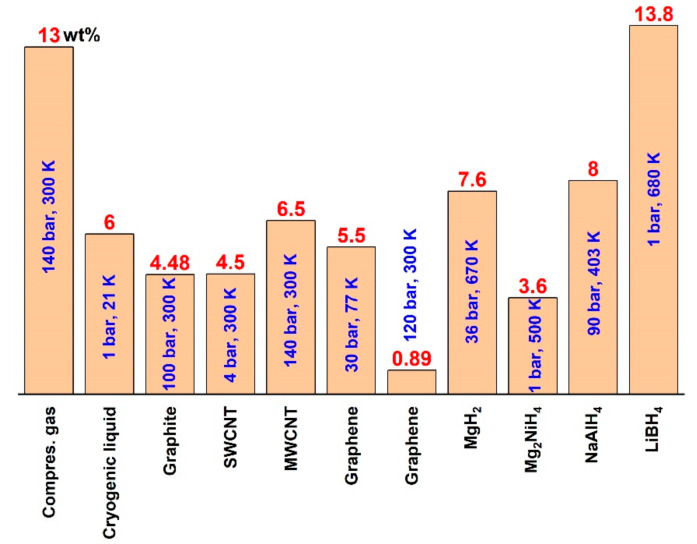
Hydrogen storage capacity (wt%) for some important materials. The kinetic conditions for hydrogen storage are shown.

Another type of metal hydride is complex hydride [16,45,46,47,48]. Today, the highest gravimetric hydrogen density is about 13.8 wt% for LiBH_4_, making this complex the ideal hydrogen storage material for vehicle transportations if the storage kinetics are improved (Figure 1) [49]. Therefore, complex hydrides fortified more studies in the field of hydrogen storage materials. Today, there is a need for discovering a new complex hydride based on lightweight metals with the highest possible gravimetric hydrogen density.

Dual-tuning impacts of the thermodynamics and kinetics for hydrogen storage materials are significant issues for the hydrogen economy, especially for the metal hydrides such as LiBH_4_ and MgH_2_. Newly developed ball milling with aerosol spraying for dual-tuning of the thermodynamics and kinetics of LiBH_4_ shows future opportunities to improve the reversible hydrogen storage properties of metal hydride-based materials in a solid state [42,50,51,52,53].

Besides metal hydride-based hydrogen storage materials, many carbon-based nanostructured materials have been considered. Experiments showed that the adsorption–desorption process is reversible, and the amount of adsorbed hydrogen is relatively low (about 3 wt%) [12,21,23,54]. Carbon nanostructures and other nanoporous materials have also been examined for hydrogen storage [23,25,55,56,57,58]. It is found that the amount of absorbed hydrogen, which is very low, increased with temperature and pressure, indicating that the mechanism of storing is of a chemical reaction nature, preferably physisorption. It is concluded that the relatively low values of gravimetric and volumetric hydrogen densities for carbon, carbon-based structures, and other porous materials are significant drawbacks.

When selecting a potential storage system, the kinetic characteristics of the hydrogen storage material must be considered. Indeed, obtaining a rapid hydrogenation–dehydrogenation process is a significant issue for many hydrogen storage materials. Therefore, the reversible hydrogen storage kinetics and capacity strongly depend on the materials’ preparation methods and applied conditions.

Inspired by the above facts, we have investigated light metal hydrides combined with a carbon-based nanostructure to obtain an excellent hydrogen storage material to assist future clean energy.

## 2. Materials and Methods

Before deposition of carbon nanotubes, a metal-supported catalyst is required. Typically, nickel or titanium metal is employed to grow fine vertical tubes on a particular substrate. Silicon is the common universally utilized substrate for CNTs growth. This research study uses an alloy consisting of two different metals as a metallic catalyst, specifically palladium and magnesium. This alloy is supposed to generate long carbon nanotubes with fibrous-like structures. The fibrous carbon nanotubes will enhance hydrogen absorption–description kinetics. Moreover, 10 nm PdMg thin alloy film was deposited on Pt substrate by co-sputtering (Univex 360, Leybold Inc., Cologne, Germany). Two high purity (99.999%) targets, Pd and Mg, were concurrently sputtered to form the alloy layer with a power of 40 and 30 watts, respectively. The base pressure was 10^−6^ mbar, the working pressure was 10^−2^ mbar, and the argon partial pressure was 90 sccm. A plasma-enhanced chemical vapor deposition (PECVD) system (EasyTube 2000, FirstNano Inc., Central Islip, NY, USA) grew carbon nanotubes on Pt substrate loaded with the PdMg catalyst. After deposition of the PdMg alloy layer on Pt substrate was accomplished, the substrate was transferred to PECVD system. The PECVD system was supplied with a graphite heater, a three zones furnace to obtain a uniform temperature throughout the substrate, a vacuum pump, and a quartz tube. The quartz tube was evacuated to a base pressure of 10^−3^ mbar. As the first step, N_2_ is purged into the quartz tube for two minutes and followed by an Ar gas for 5 min. Hydrogen gas with a flowing rate of 90 sccm was introduced into the quartz tube, and the oven was heated to 500 °C with a heating rate of 20 °C/min. The system was halted at these conditions for 30 min. After this time, the furnace was adjusted to reach 650 °C with a 5 °C/min heating rate. At 650 °C, NH_3_ (90 sccm) was injected into the quartz tube for plasma initialization (50 W). After plasma stabilization at 60 W, a high purity C_2_H_2_ gas (25 sccm) was injected into the quartz tube to grow the carbon nanotubes. The oven cooled down to room temperature under an Ar atmosphere when the preferred reaction time was achieved. The obtained carbon nanotubes were transferred to the sputtering chamber to deposit the PdMg thin alloy top layer with the conditions mentioned earlier.

The obtained nanocomposite samples were examined by using a field emission scanning electron microscope (ZEISS Sigma FESEM, Carl Zeiss GmbH, Oberkochen, Germany), XRD (Ultima IV, Rigaku Corporation, Tokyo, Japan), and a Raman spectroscope (HR800, HORIBA Europe GmbH, Oberursel, Germany).

Electrochemical hydrogen storage was performed using a standard three-electrode cell connected to an electrochemical workstation (PGSTAT302N, Metrohm AG, Herisau, Switzerland). All measurements have been done at an ambient temperature and pressure. A commercial platinum sheet with a total surface area of about 1 cm^2^ was used as a counter electrode. The thin film sample was attached (free substrate surface) to a platinum wire using a conductive adhesive to prepare the working electrode. A chemically inert insulating varnish was applied to the contact and edges of the film to protect them from the electrolyte. The active surface area of the working electrode was about 2 cm^2^. The charged–discharged experiment was performed at a constant current of ±150 mA under a cut-off potential of −0.5 V vs. Ag/AgCl electrode. To account for the high-rate discharge-ability (HRD), the working electrode was discharged at different constant currents (A = 25, 50, 75, 100, and 125 mA/g). HDR is defined as HRD = C_A_/C_25_ × 100%, and C_A_ is the maximum capacity at the selected current density.

## 3. Results

**Figure 2:**
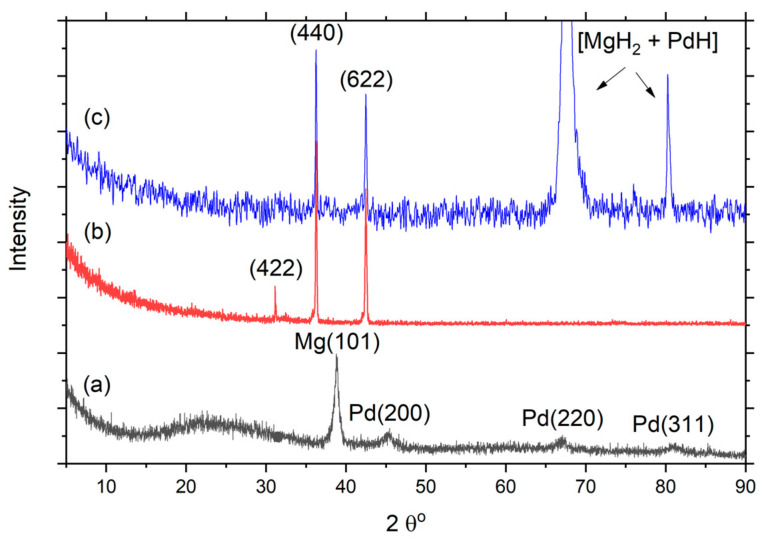
XRD pattern: (**a**) PdMg binary alloy deposited on a glass substrate, and (**b**,**c**) graphene/*f*-MWCNT nanocomposite decorated with PdMg alloy nanoparticles deposited on Pt substrate before and after hydrogenation, respectively.

Figure 2 shows the XRD pattern of graphene/*f*-MWCNT composite film decorated with top PdMg alloy layer (G/*f*-MWCNT@PdMg). The XRD pattern in Figure 2b displayed three diffraction peaks, two intense diffraction peaks around 36° and 42° and a low intensity diffraction peak around 31°. These peaks are assigned to (440), (533), and (422) diffraction plans of typical C60. Fullerenes and carbon nanotubes are allotropes of carbon categorized by a void structure and remarkable physical properties. The void sites are advantageous for hydrogen adsorption [54]. This result indicates that graphene and *f*-MWCNTs are well graphitized. There is no indication of any carbon impurities. The metal particles of the PdMg top thin layer are not recognized due to their nanoparticle size of the alloy. In fact, due to the surface nature of carbon nanotubes, the metal particles of PdMg alloy are dispersed and implanted into the composite structure instead of forming a continuous top thin layer. Metal nanoparticles, when distributed over carbon nanostructure materials, demonstrate improved storage capacities and enhanced adsorption–desorption kinetics [59,60].

The bottom XRD pattern in Figure 2a is the typical for PdMg alloy deposited on glass substate. The pattern exhibits one intense peak around 38.81° and three other small peaks around 45.32°, 66.98°, and 80.96°. The first intense peak is corresponding to a hexagonal Mg structure (JCPDS card no. 00-350-821), while the other three smaller peaks are corresponding to an fcc Pd structure (JCPDS card no. 00-152-2945).

It is noted that the position of the first peak is shifted toward a larger angle in comparison to bulk Mg, while the other three peaks shifted toward smaller angles. The calculated lattice parameter of Pd is 3.96 Å, which is larger than the corresponding bulk cubic structure (a = 3.89 Å). This information suggested that the cell volume of Mg is contracted due to the presence of Pd atoms, while the cell volume of Pd is enlarged due to the presence of Mg atoms. Hence, it is concluded that the deposited PdMg film contains two mixed phases: a host Mg phase with dopped Pd atoms, and a host Pd phase with dopped Mg atoms. More information is presented in Table 1.

XRD is not a very useful tool when distinguishing between graphene and carbon nanotube is required. Raman spectroscopy is a very effective tool in such a situation. Usually, Raman spectra for graphene or nanotubes include three different bands: G-band, D-band, and 2D band. The G-band arises from the vibration of sp2 carbon atoms. The D-band is known as the disorder band and is related to structural defects of sp3 carbon. The band is strong in carbon nanotubes and typically very weak in graphene. The shape of the 2D band is very important. A single symmetric peak means one graphene layer, while more graphene layers produce an asymmetric 2D peak [61].

**Figure 3:**
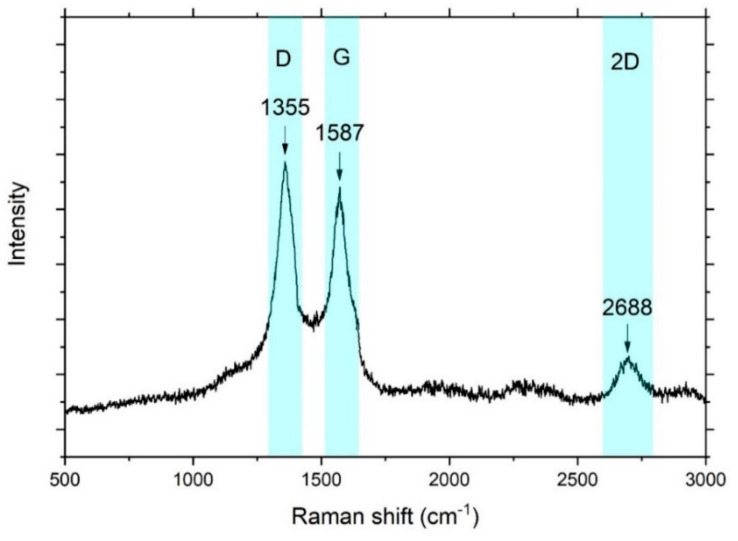
Raman spectra of graphene/*f*-MWCNT nanocomposite @PdMg sample.

Figure 3 shows Raman spectra of the G/*f*-MWCNT@PdMg sample. The figure exhibits three bands located at 1355 cm^−1^, 1587 cm^−1^, and 2688 cm^−1^ corresponding to D-, G-, and 2D-band, respectively. Using a 2D band, the difference among graphene, nanotubes, and graphene-carbon nanotube composite can be recognized. Nanotubes always display intense 2D bands, while graphene always shows very weak intensity. Here, the 2D band is seen with reasonable intensity, indicating that a mixed phase of graphene and carbon nanotubes is successfully formed.

**Figure 4:**
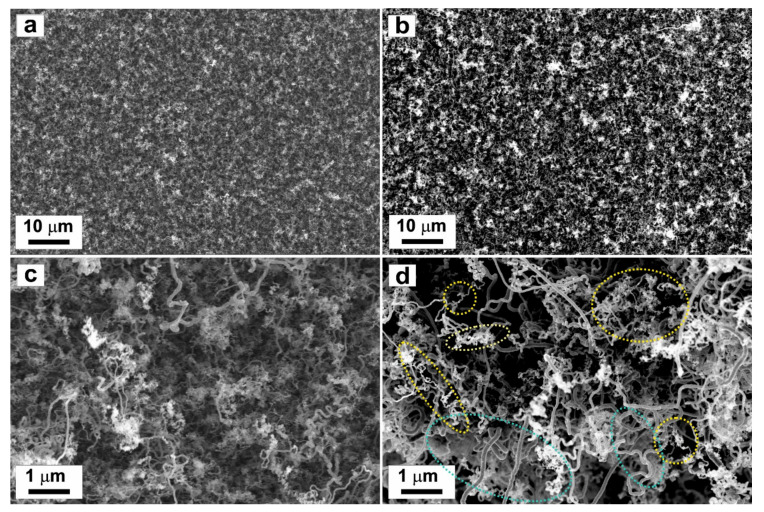
High-resolution SEM images of (**a**,**c**) graphene/*f*-MWCNT nanocomposite and (**b**,**d**) graphene/*f*-MWCNT nanocomposite decorated with PdMg alloy nanoparticles deposited on Pt substrate. Yellow ovals highlight the decorative clusters of PdMg nanoparticles.

The surface morphology of the prepared nanocomposite is presented in Figure 4. The prepared composite without PdMg alloy nanoparticles is shown in Figure 4a,c, while the composite decorated with PdMg nanoparticles is given in Figure 4b,d. The PdMg nanoparticles are dispersed into the bulk of the composite film as indicated in the figure by yellow shapes. All images display the fibrous structure of carbon nanotubes. There are some transparent layers of graphene shown in Figure 4d by green shapes. These SEM images with the information deduced from Raman spectra confirm that the graphene/f-MWCNT nanocomposite @PdMg alloy nanoparticles are successfully formed. The composite contains many voids and hollow sites, which are favorable for high-capacity hydrogen absorption characteristics. The presence of PdMg nanoparticles is expected to increase storage capacity and enhance reaction kinetics.

**Figure 5:**
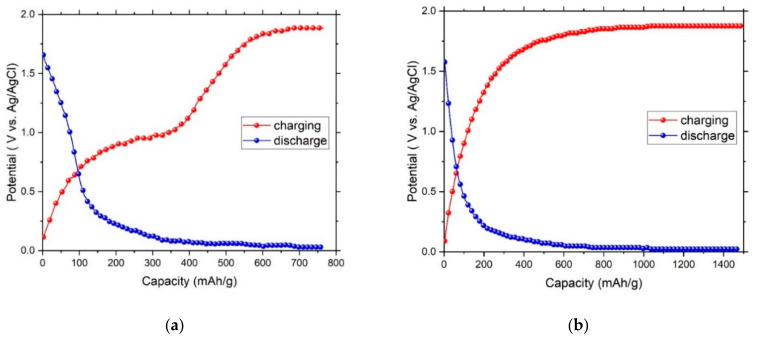
The charge–discharge curves in 3M KOH electrolyte for (**a**) G/f-MWCNT electrode; (**b**) G/*f*-MWCNT@PdMg electrode.

The charge and discharge curves of G/*f*-MWCNT and G/*f*-MWCNT@PdMg nanocomposites are shown in Figure 5a,b, respectively. A discharge capacity of 765 mAh/g is obtained in the G/*f*-MWCNT electrode corresponding to ∼2.86 wt% hydrogen, while the best discharge capacity of G/*f*-MWCNT@PdMg electrode is 1478 mAh/g corresponding to ∼5.53 wt% hydrogen content. It has been stated that the aligned carbon nanotubes exhibit a better hydrogen absorption capacity compared to non-aligned nanotube [62]. Here, the G/*f*-MWCNT@PdMg sample shows a higher absorption capacity than the sample without PdMg nanoparticles. In fact, the obtained capacity for graphene/carbon nanotube composite decorated with PdMg nanoparticles displays a higher absorption capacity than other carbon nanotubes materials found in the literature considering the film structure of the sample [12,20,21,23,24,36,62]. After several cycles, the samples almost maintain their capacities (the figure is not shown here).

The discharge capacities with appropriate cyclic stabilities and the dehydrogenation activation properties are the key parameters to judge for good hydrogen storage materials. Due to the presence of many suitable absorption sites for hydrogen storage, the carbon nanotubes possessed a high theoretical storage capacity exceeding 2500 mAh/g depending on structure, morphology, and defect concentration; however, the maximum experimental storage capacities are still frustrating [63,64].

**Figure 6:**
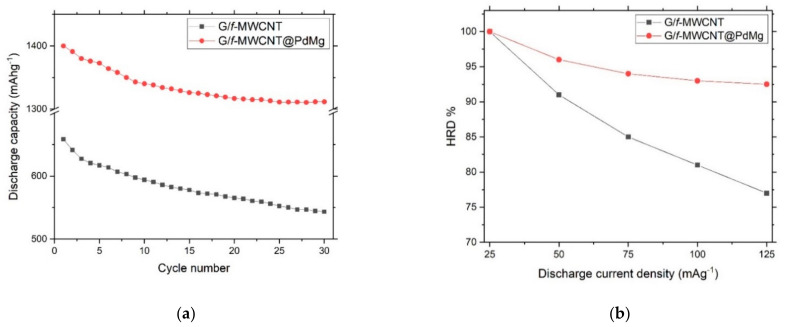
(**a**) Discharge capacities of the G/*f*-MWCNT and G/*f*-MWCNT@PdMg nanocomposites at a current density of 25 mA/g: G/*f*-MWCNT electrode; (**b**) high-rate discharge-ability of the G/*f*-MWCNT and G/*f*-MWCNT@PdMg nanocomposites.

Figure 6a shows the discharge capacities (30 cycles) at a current density of 25 mA/g for G/*f*-MWCNT and G/*f*-MWCNT@PdMg nanocomposites. A noticeable enhancement of discharge capacity and cyclic stability is obtained for the G/*f*-MWCNT@PdMg sample in comparison to the G/*f*-MWCNT sample. Another important factor for a suitable candidate for storage materials is its ability to sustain the discharge performance at a high current density. The HRD for G/*f*-MWCNT and G/*f*-MWCNT@PdMg nanocomposites at different discharge current densities is shown in Figure 6b.

The improved HRD performance of the G/*f*-MWCNT@PdMg sample compared to the G/*f*-MWCNT can be explained as follows: the small sizes of PdMg nanoparticles for the composite sample diminish the diffusion lengths for hydrogen from the absorbed/adsorbed sites to the electrode/electrolyte interface. This assists in the contact between alloy and electrolyte, and provides quick charge transfer networks within the sample.

**Figure 7:**
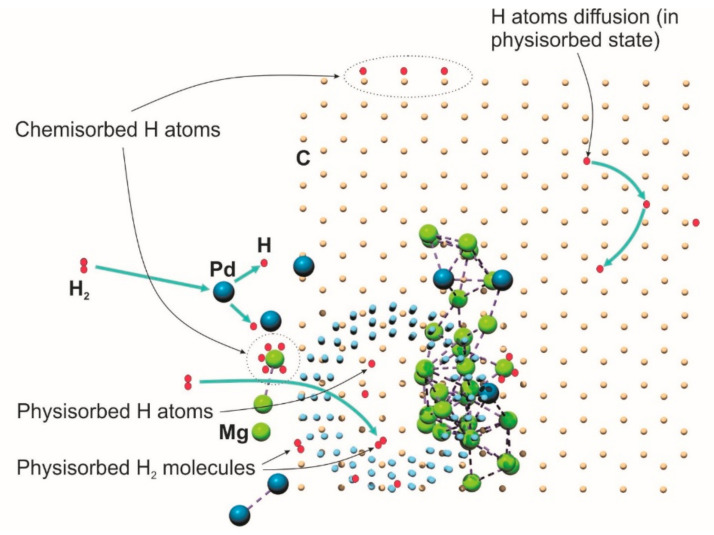
Schematic illustration of hydrogen absorption in G/f-MWCNT@PdMg sample.

Figure 7 shows an illustration of the hydrogen absorption process in the sample. Hydrogen is stored in materials through two different mechanisms: absorption when hydrogen molecules are stored directly within the free spaces in the material, and adsorption when hydrogen atoms bonded to the surface of the material. Generally, metal hydride is formed through a sequence of stages explained as follows: physisorption (Van der Waals attractive forces between the metal and hydrogen molecules catches in an accessible volume close to the metal) → dissociation of the hydrogen molecules at the metal surface (the metal catalyst, for example; Pd assists this process) → chemisorption, i.e., the formation of the hydrogen bond at the metal surface → α-phase formation (hydrogen occupies interstitial sites) → β-phase (new physicochemical properties arise with the increasing concentration of hydrogen in the metal crystal lattice) → vanishing of α-phase.

Therefore, the nanosize of PdMg particles allows more hydrogen dissociation sites and shortened hydrogen diffusion pathways that improve storage kinetics [65,66,67,68]. In addition, a larger surface area increases the number of atoms at the grain boundaries, which in turn enhances hydrogen diffusion rates. On the other hand, the large surface area of graphene/nanotube as well as the large interstitial volumes within the nanofibrous structure create huge hydrogen storage sites. Figure 2c shows the XRD pattern of the G/*f*-MWCNT@PdMg sample after hydrogenation. The pattern exhibits two new peaks (located at 67.68° and 80.2°) in comparison to the sample before hydrogenation in Figure 2b. These new peaks arise from tetragonal MgH_2_ (JCPDS card no. 00-089-7887) and fcc PdH (JCPDS card no. 00-065-0557) phases. As mentioned before, the deposited PdMg film contains two mixed phases, one of which is the host Pd phase with dopped Mg atoms. This phase is characterized by the high hydrogen storage capacity. Most Mg atoms are surrounded by Pd atoms. This arrangement allows Mg atoms to absorb more hydrogen, and also creates more hydrogen diffusion paths to graphene and nanotube structures with a high hydrogen diffusion rate. The catalytic nature of palladium enhanced the absorption kinetics, and the presence of magnesium increased the storage capacity of hydrogen. The fibrous structure of the nanotubes created room for further absorption of molecular hydrogen. The graphene layers increased the surface area for atomic hydrogen absorption. Nanoparticles made of metal alloy embossed with a carbon nanostructure appear to be a viable material for hydrogen storage applications.

## 4. Conclusions

This work investigates the hydrogen storage capacity of graphene/multiwall carbon nanotubes decorated with PdMg alloy nanoparticles (G/*f*-MWCNT@PdMg). It was found that the presence of PdMg nanoparticles increased the hydrogen absorption capacity by 53% compared to the sample without PdMg decorated nanoparticles. The obtained hydrogen capacities for both samples with and without PdMg nanoparticles are 2.86 wt% and 5.53 wt%, respectively. This higher value of the hydrogen storage capacity was ascribed to the fact that PdMg nanoparticles act as active reaction sites for molecular and atomic hydrogen absorption and desorption. The catalytic nature of Pd metal enhanced the absorption kinetics, and the presence of Mg increased the hydrogen storage capacity.

Furthermore, the fibrous structure of the nanotubes created rooms for more molecular hydrogen absorption. Moreover, the graphene layers increased the surface area for atomic hydrogen absorption due to their catalytic nature. Thus, the carbon nanostructure decorated with PdMg alloy nanoparticles appears to be a viable material for hydrogen storage applications.

## Figures and Tables

**Table 1:** Structure parameters of PdMg alloy.

Phase	2θ°		d Spacing, Å		∆θ°	∆d, Å	Lattice Parameters, Å		Cell Volume, Å3	
	Film	Bulk	Film	Bulk			Film	Bulk	Film	Bulk
Mg (101)	38.81	36.62	2.318	2.450	2.19	−0.132	-	-		
Pd (200)	45.32	46.66	1.998	1.945	−1.32	0.053	3.99	3.89	63.52	58.87
Pd (220)	66.98	68.12	1.396	1.375	−1.14	0.021	3.95		61.63	
Pd (311)	80.96	82.09	1.186	1.173	−1.14	0.014	3.94		61.16	

## Data Availability

No data.
